# Acute Abdomen Secondary to Granulomatous Appendicitis: A Rare Case of Complicated Appendicitis

**DOI:** 10.7759/cureus.23247

**Published:** 2022-03-17

**Authors:** Tala Abedalqader, Mohamad Bakir, Fares AlJohani, Talal Altahan, Sadiq M Amer, Sami Almustanyir

**Affiliations:** 1 Medicine, Alfaisal University, Riyadh, SAU; 2 Internal Medicine, Prince Mohammed bin Abdulaziz Hospital, Riyadh, SAU; 3 Clinical Affairs, Prince Mohammed bin Abdulaziz Hospital, Riyadh, SAU; 4 Pathology, Prince Mohammed bin Abdulaziz Hospital, Riyadh, SAU; 5 Research, Ministry of Health, Riyadh, SAU

**Keywords:** case report, surgery, inflammatory bowel disease, acute abdomen, granulomatous appendicitis, granulomatous, appendicitis

## Abstract

Acute appendicitis is one of the most common surgical presentations seen in the emergency department, usually presenting as a case of fever, anorexia, and abdominal pain. Curative treatment is an appendectomy with histological examination of the surgical specimen to diagnose the subtypes or causes of appendicitis. One of these subtypes, granulomatous appendicitis, is an uncommon form of appendicitis. This condition can be caused by a multitude of mechanisms, including tuberculosis infections, parasitic infections, fungal infections, mechanical obstruction, or systemic diseases such as Crohn's disease, sarcoidosis, among others. Investigations and management should be tailored according to the histologic findings, and patient follow-up should be advised.

## Introduction

Acute appendicitis is an acute inflammation of the vermiform appendix, most likely occurring due to obstruction of the lumen of the appendix by a fecalith, normal stool, infective agents, or lymphoid hyperplasia. It typically presents as acute pain in the mid-abdominal area that later localizes to the right lower quadrant, associated with fever, anorexia, nausea, vomiting, and leukocytosis [1]. Granulomatous appendicitis is an uncommon subtype of appendicitis and can be caused by a variety of conditions including systemic disorders such as Crohn’s disease and sarcoidosis, as well as infections such as *Mycobacterium tuberculosis*, *Yersinia pseudotuberculosis*, parasites, and fungi [2]. We present a case of a 20-year-old male with appendicitis that was identified as granulomatous appendicitis on histopathology of the specimen and discuss the correlation between clinical and radiological findings.

## Case presentation

A 20-year-old male patient, with a known case of diabetes mellitus type two being treated with metformin, presented to the emergency department at our hospital with a three-day history of right lower quadrant abdominal pain. The pain began in the periumbilical region and then localized to the right lower quadrant. It was associated with fever, vomiting, anorexia, and dysuria. No changes in bowel movements, steatorrhea, jaundice, or scleral icterus were observed, nor were there any changes in urine color or output, melena, hematochezia, weight loss, night sweats, or pale stools. Additionally, the patient did not have any shortness of breath, cough, changes in vision, or skin changes. Past medical history was significant only for diabetes mellitus type two, controlled with metformin. Past surgical history included vertebral fixation two years prior. Family history was unremarkable. The patient was a non-smoker who ate a regular diet rich in carbohydrates, fiber, and protein, and led a sedentary lifestyle.

On physical examination, the patient appeared in distress due to pain but was vitally stable. Abdominal examination was remarkable for right lower quadrant tenderness with positive Rovsing sign and positive rebound tenderness without rigidity or palpable masses. Psoas sign was negative. Oral examination showed moist mucous membranes, good hygiene, and no ulcerations. No scleral icterus or pallor was noted. Chest examination showed equal bilateral air entry with no rales or wheezes. Cardiovascular examination showed normal S1, S2 with no added sounds. No skin changes/rashes were observed. No peripheral edema was present. Laboratory investigations on admission showed normal WBC, neutrophil and lymphocyte counts, and C-reactive protein levels were within the reference. Of note, the total bilirubin level was elevated, and prothrombin time (PT) was prolonged. Otherwise, complete blood count, coagulation profile, hepatic profile, and renal profile parameters were within normal limits. Hepatitis B, C and HIV serologies were negative (Table 1).

**Table 1:** Lab values throughout the admission

Investigation	Value	Reference Range
Red blood cell (RBC)	5.07 x10^9^/L	4.7-6.1 x 10^9^/L
Hemoglobin	16.10 g/L	14-18 g/dL (men)
Platelet Count	208 x10^9^/L	150-400 x 10^9^/L
White blood cells (WBC)	7.25 x10^9^/L	4-10 x 10^9^/L
Neutrophils %	61.80%	55-70%
Lymphocytes %	29.80%	20–40%
Monocytes %	7.40%	2–8%
Eosinophils %	0.82%	1–4%
Basophils %	0.1%	0.5-1%
C-reactive protein	0.15 mg/dL	0-1 mg/dL
Creatinine	74.80 μmol/L	44-97 μmol/L
Urea level	6.8 mmol/L	3.6-7.1 mmol/L
Sodium	140 mmol/L	135-147 mmol/L
Potassium	4.1 mmol/L	3.5-5.0 mmol/L
Chloride	110 mmol/L	98-106 mmol/L
Albumin	4.7 g/dL	3.5 to 5.5 g/dL
Aspartate aminotransferase (AST)	27 U/L	0-35 U/L
Alanine transaminase (ALT)	21 U/L	4-36 U/L
Total bilirubin	24.0 μmol/L	5.1-17 μmol/L
Prothrombin time (PT)	17.4 seconds	11.0-12.5 seconds
International normalized ratio (INR)	1.28	0.8-1.1
Activated partial thromboplastin time (aPTT)	41.3 seconds	30-40 seconds
Hepatitis B surface antigen	>1000.00	Negative
Hepatitis B surface antibodies	High Reactive	Negative; >10 U/mL protective
Hepatitis B core antibodies	Nonreactive	Negative
HIV antigen/Antibody	Nonreactive	Negative
Hepatitis C antibody	Nonreactive	Negative

**Figure 1:**
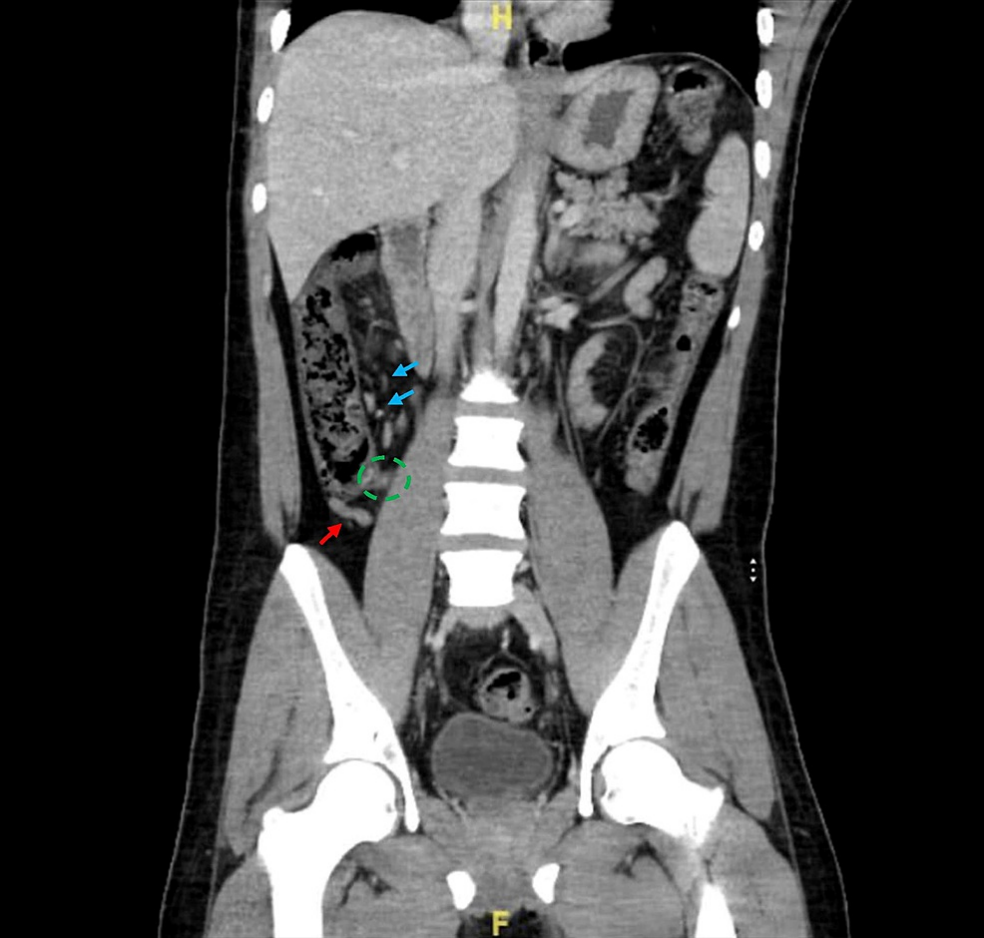
CT of the abdomen with contrast The appendix (red arrow) is seen at the right iliac fossa with a normal caliber of about 7mm, minimal surrounding fat stranding (green dashed circle), and small mesenteric lymph nodes (blue arrows), consistent with an early acute appendicitis diagnosis.

A CT scan of the abdomen showed findings consistent with a diagnosis of early acute appendicitis. The appendix was seen at the right iliac fossa with a normal caliber of 7 mm, with minimal surrounding fat stranding and small mesenteric lymph nodes. Solid abdominal organs appeared unremarkable, and no renal stones, free fluid, or free air were noted (Figure 1).

The patient was admitted as a case of acute appendicitis, for appendectomy. He was kept nothing by mouth (NPO) for eight hours before surgery. Intravenous (IV) fluids and prophylactic antibiotics were initiated. Paracetamol was administered for analgesia. A COVID-19 polymerase chain reaction (PCR) test was performed, and the result was negative. Laparoscopic appendectomy was performed under general anesthesia with no complications, and the surgical specimen was sent for histopathology. Thromboprophylaxis using heparin was started following surgery. 

**Figure 2:**
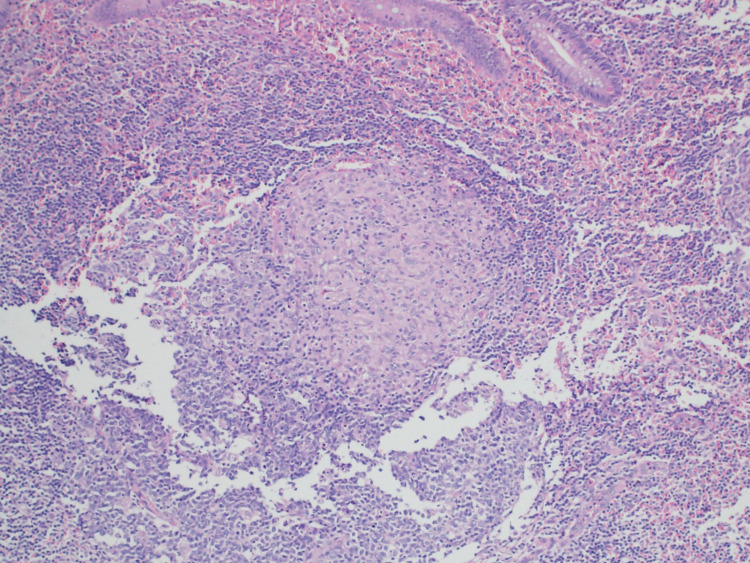
Histology of surgical specimen: Epithelioid granuloma Sections represent appendiceal tissue showing multiple granulomas in the submucosa, muscularis propria, and subserosa in a background of neutrophilic infiltrate of the mucosa. Most of these granulomas are ill-defined. However, one is a well-defined tight aggregate of epithelioid histiocytes. No firm evidence of inflammatory bowel disease is seen in the examined material.

The surgical specimen was received in formalin labelled as “appendix”, consisting of an intact appendix measuring 10 cm x 1 cm with a congested outer surface and a focally dilated lumen filled with hemorrhagic material. Under histological examination, granulomatous inflammation was visualized on a background of acute appendicitis. Sections of appendiceal tissue showed multiple granulomas in the submucosa, muscularis propria, and subserosa in a background of neutrophilic infiltrate of the mucosa. Most of the granulomas were ill-defined except for one granuloma that was well-demarcated with an aggregate of epithelioid histiocytes. Accompanying giant cell reaction was noted but no central necrosis was seen. No firm evidence of inflammatory bowel disease was noted in the examined material (Figure 2). The periodic acid-Schiff (PAS) and Ziehl-Neelsen (ZN) stains for fungal elements as well as the acid-fast bacillus (AFB) stain, were all negative.

Post-operatively, the patient had no complications, was tolerating orally with no nausea or vomiting, and was mobilizing well. The surgical scar was clean with no discharge or erythema. The patient was discharged after three days of hospitalization with a scheduled follow-up. On follow-up two weeks later, central/laparoscopy scars showed good healing with no sign of infection of surgical wounds.

## Discussion

Granulomatous appendicitis is a rare inflammatory condition affecting the appendix, with a reported frequency of less than 2%. The pathogenesis of this condition encompasses a wide range of possible etiologies including infectious (38%) or non-infectious conditions (62%). Thus, it is important to identify granulomatous appendicitis and investigate the underlying etiologies after surgery using appropriate examinations.

Infectious causes of granulomatous appendicitis can include *Yersinia* species, *Mycobacterium tuberculosis*, *Enterobius vermicularis*, and *Actinomyces* species. Non-infectious causes encompass diverticulitis, foreign body granulomatous reactions, tumors, sarcoidosis, and inflammatory bowel disease (Crohn's disease) [3]. Many reports describe the association of granulomatous inflammation of the appendix and Crohn's disease, which can manifest in one of two types: (1) appendiceal Crohn disease, in which inflammation is limited to the appendix; and (2) appendiceal involvement of Crohn's disease, in which inflammation spreads to the appendix in patients with ileal/cecal involvement [4]. Delayed appendectomy performed after conservative management of a perforated appendix can also be associated with marked granulomatous features. Idiopathic granulomatous appendicitis is a very rare disease.

The presentation of granulomatous appendicitis mimics the presentation of acute appendicitis with an ordinary range of inflammation. Patients typically present with fever, nausea, vomiting, anorexia, abdominal pain (periumbilical initially localizing to the right inferior quadrant), and leukocytosis. Physical examination and personal history are important parameters in the diagnosis of granulomatous appendicitis. Specifically, patients should be questioned about clinical features, risk factors, and exposure to infectious causes as well as symptomology of non-infectious causes such as weight loss, fever, night sweats, anorexia, and others. Additionally, a detailed history should be taken to rule out sarcoidosis (pulmonary symptoms, skin changes, uveitis, along with others) and inflammatory bowel disease (diarrhea bloody/watery, abdominal pain, nutritional deficiency signs, extraintestinal manifestations, etc.). Physical examination should be tailored to identifying signs specific to acute appendicitis, and other features that could indicate underlying diseases, as mentioned above, should be noted.

The confirmatory diagnosis of granulomatous appendicitis is made by pathology, following appendectomy and examination of the specimen under microscopy. Macroscopically, appendicular inflammation is classified as “normal”, “inflamed”, “phlegmon”, “gangrenous”, or “perforated”. Other gross characteristics, such as nodular appearance, abscess-like appearance, or the presence of adhesions, could help guide the diagnosis of granulomatous inflammation [5]. Histopathology is characterized by the presence of non-necrotizing granulomas consisting of epithelioid and multinucleated giant cells as well as inflammatory infiltrates [6]. Additional diagnostic investigations should be aimed at identifying underlying etiologies. For instance, specific diagnostic tests for appendicular tuberculosis include acid-fast stain (AFB), tissue culture, tissue PCR, purified protein derivative (PPD) test, and interferon-gamma release assays (IGRAs). Periodic acid-Schiff (PAS) stain can be used for the detection of fungal elements. Furthermore, colonoscopy, anti-saccharomyces cerevisiae antibody (ASCAs), and anti-neutrophilic cytoplasmic antibody (ANCAs) tests can be used in the differential diagnosis of cases with histopathological findings indicating Crohn’s disease [7]. Radiologic investigations, such as CT scans of the chest/abdomen, chest X-ray, and others can further aid in diagnosis.

Although appendectomy is a curative treatment for granulomatous appendicitis, further treatment should be administered to the patient based on the underlying mechanism of the disease. Regular follow-ups should be advised to the patient to monitor disease progression and treatment effectiveness. In addition, follow-ups could help in the detection and management of complications associated with infectious or systemic diseases associated with granulomatous appendicitis. 

## Conclusions

Granulomatous appendicitis is an uncommon subtype of appendicitis with multiple possible etiologies including infectious and systemic diseases. Surgical excision of the inflamed appendix is the curative treatment for this condition, and confirmatory diagnosis is made through histopathological examination of the surgical specimen. Additional histological, radiologic, and laboratory investigations should be performed to identify the underlying cause of the granulomatous inflammation. Treatment should be tailored accordingly to slow progression and prevent recurrence and complications of the underlying causative disease.
